# Concurrent Varicella-Zoster Virus Reactivation and Recurrent Herpes Simplex Virus Infection in the C2 Dermatome With Varicella-Zoster Virus Encephalitis: A Case Report and Review of the Literature

**DOI:** 10.7759/cureus.67479

**Published:** 2024-08-22

**Authors:** Morgan A Hatlovic, Madison Anzelc, Matthew Franklin

**Affiliations:** 1 Dermatology, Lake Erie College of Osteopathic Medicine, Bradenton, USA; 2 Dermatology, OhioHealth Riverside Methodist Hospital, Columbus, USA

**Keywords:** vesicles, concurrent, vzv encephalitis, varicella-zoster virus, herpes simplex virus

## Abstract

Herpes simplex virus (HSV) and varicella-zoster virus (VZV) are common viruses that are present in the general population. However, it is uncommon for both viruses to coincide at the same time and location. These viruses infect the nervous system to establish latency and have been associated with neurological disorders. We discuss a case of co-occurring VZV reactivation and recurrent HSV infection with subsequent VZV encephalitis following an insult to the neurologic system.

## Introduction

Herpes simplex virus (HSV) and varicella-zoster virus (VZV) are part of the herpesvirus family. These viruses are unique in how they can enter the dorsal root ganglia through sensory nerve pathways and remain inactive in a latent state. Despite sharing a neural tropism for sensory ganglia, HSV and VZV maintain latency in different cell types. HSV establishes latency in neurons while VZV remains in satellite cells surrounding neurons [1]. Although HSV and VZV can lie dormant in cells of the same ganglion, they may be reactivated by different triggers. Reactivation for VZV is more commonly provoked by trauma and X-ray imaging, while HSV is more likely to recur due to stress, fever, menstruation, UV light exposure, immunosuppression, or direct trauma to the face [2-5]. The clinical presentation of HSV is vesicles with clear fluid that progress to pustules, which eventually rupture, ulcerate, and form a scab. VZV presents as vesicles that become pustular, break open, and crust [3,4].

The age and frequency at which herpesviruses undergo reactivation often differ depending on the virus. HSV tends to resurface from latency at a younger age, usually before 40, and can subsequently present itself multiple times throughout a patient's life. In contrast, VZV typically reemerges in older individuals usually after 60 and typically only once, likely due to a decline in cellular immunity as an individual ages [1,2,5-7]. Supporting this theory is a study that showed a decline in cellular immunity to VZV starting as early as 40 years old [8].

Despite both viruses being common in the general population, it is rare to have simultaneous presentations of both HSV and VZV due to their differences in reactivation [2]. Although extremely unlikely, it is even possible for them to co-occur in the same region of the skin since HSV and VZV can remain dormant in the same ganglion [5]. It is theorized that concurrent reactivation happens because both viruses share the same DNA polymerase enzyme. When one virus reactivates and induces this enzyme, it leads to heightened enzyme activity, thereby triggering the reactivation of the other virus simultaneously [5].

We present a case of a patient with concurrent herpes zoster and recurrent HSV infection in the C2 dermatome, further complicated by VZV encephalitis.

## Case presentation

The patient is a 61-year-old HIV-negative man with glucose-6-phosphate dehydrogenase (G6PD) deficiency and end-stage renal disease on hemodialysis who presented to a local hospital emergency department for urgent dialysis. While undergoing dialysis, he became unresponsive and was found to have an acute intracerebral hemorrhage (ICH). The hospital course was complicated by sepsis secondary to a hemodialysis catheter infection with methicillin-resistant Staphylococcus aureus infection, which required removal of the catheter and intravenous antibiotics. The dermatology service was consulted two weeks after admission to evaluate for possible bullous impetigo near the site of hemodialysis catheter removal. 

**Figure 1:**
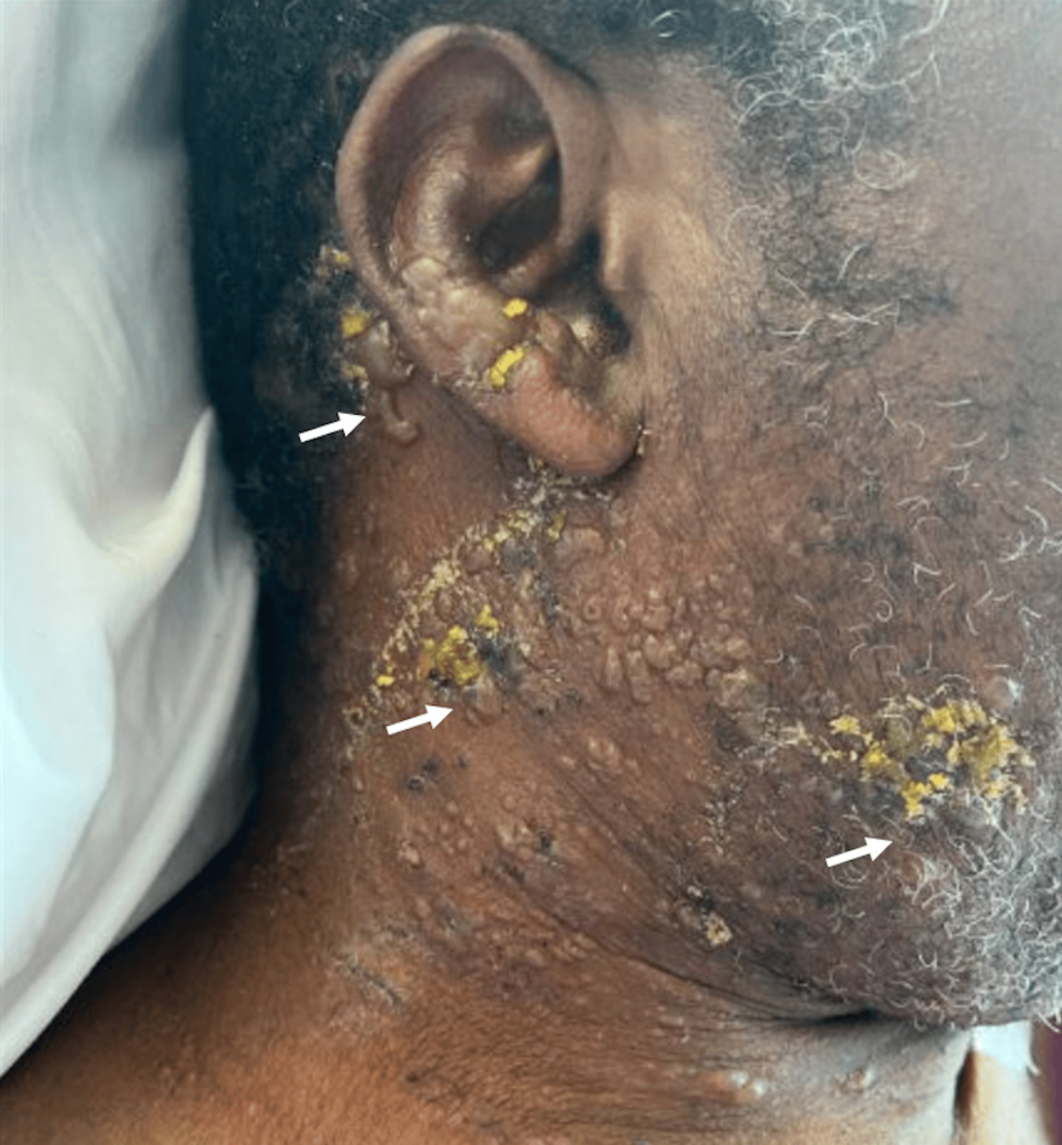
Vesicles with overlying honey-colored crust. White solid arrows point to clusters of vesicles.

Upon evaluation, the patient had numerous vesicles with an overlying honey-colored crust (Figure 1, white solid arrows) on the right cheek, right post-auricular skin, and right anterior neck. There was hemorrhagic crusting on the ipsilateral aspect of the upper and lower lips. There were also two punched-out erosions noted on the right posterior neck and right chin. The eruption did not cross the midline. VZV and HSV polymerase chain reaction (PCR) swabs were collected from a vesicle on the right lateral cheek, both of which returned positive results. The results were positive for HSV-1 and negative for HSV-2. The patient was diagnosed with co-occurring VZV reactivation and recurrent HSV infections with impetiginization. He was started on intravenous acyclovir at a renal-adjusted dose of five mg/kg every 24 hours for a seven-day course, as well as given topical mupirocin ointment to apply to areas with superficial crusting. 

Due to seizure-like activity and continued encephalopathy without worsening of the ICH on neuroimaging, neurology was consulted. An electroencephalogram (EEG) was performed with evidence of generalized periodic discharges and moderate to severe slowing of the background activity, consistent with diffuse encephalopathy. With evidence of facial VZV reactivation and recurrent HSV infection, neurology was concerned about viral encephalitis and performed a lumbar puncture. Cerebrospinal fluid studies were remarkable for elevated protein, low glucose, and VZV positivity by PCR. In conclusion, the patient was diagnosed with VZV encephalitis. Infectious disease extended the acyclovir course from seven to 10 days and placed the patient in airborne precautions. He was discharged two months later after a very complicated hospital course and a significant number of social issues delaying safe discharge. 

## Discussion

Concurrent HSV and recurrent VZV infection is an uncommon clinical presentation. It is even rarer to see this coinfection in the same dermatome, due to differences in the rate, age, timing, and triggers of reactivation that differ between HSV and VZV [2,5,6]. Two retrospective studies have been conducted to identify the number of patients who present with concurrent HSV and VZV. Giehl et al. studied 1,718 immunocompetent patients with suspicious skin lesions by utilizing PCR to test for simultaneous HSV and VZV infection at the same anatomic location [6]. Results revealed only 1.2% of patients, or 20 specimens, had simultaneous infection [6]. Dhiman et al. performed an eight-year study where specimens were collected from the same anatomic site on the same date, looking for dual HSV-VZV positive specimens [9]. Of 30,305 specimens, 108 samples or 1.3% were positive for coinfection. This study found their results comparable to the study by Giehl et al., showing the coinfection rate to be very uncommon at less than 1.4% in both studies [6,9]. 

Due to the rarity of this clinical presentation, little evidence exists about the trigger for the simultaneous reactivation of herpes zoster and herpes simplex viruses. Our case involves underlying chronic kidney disease, which can affect the immune system potentially leading to infections. Therefore, this underlying condition can lead to immune system compromise [10]. Together with our case involving urgent dialysis, this should be considered as a potential trigger for immune system dysfunction leading to infection. Additionally, since the clinical course was complicated with subsequent intracerebral hemorrhage after the patient became unresponsive during dialysis, followed by sepsis secondary to hemodialysis catheter infection, it is difficult to pinpoint one specific trigger for concurrent herpes zoster and recurrent herpes simplex infection. However, both viruses share a tropism for dormancy in neural structures, so a common neurologic trigger is plausible [2-4]. We believe the ICH in our case may have been the trigger for the reactivation of both herpes viruses due to other cases in the literature.

There are other reported cases with neurologic-related triggers and simultaneous herpes zoster and herpes simplex reactivation. A case from Hafelfinger et al. describes simultaneous HSV-1 and VZV reactivation in an 81-year-old immunocompetent patient after head trauma [11]. The patient sustained minor head trauma and later presented with vesicles on the forehead that tested positive for VZV and HSV-1 [11]. Another case was reported in an 84-year-old immunocompetent patient following a traumatic lumbar spine fracture at L2-L3. Skin lesions in the right V2 dermatome tested positive for VZV and HSV-1. Specifically, facial vesicles tested positive for VZV, and hard palate cultures tested positive for HSV-1 [2]. These cases, along with our case report involving intracerebral hemorrhage, support trauma to the central nervous system as a plausible triggering event leading to recurrent HSV infection and concurrent VZV reactivation [2,11]. 

Neurologic complications such as encephalitis following VZV infection have been described in the literature. Myelitis and small-vessel encephalitis are primarily seen with immunocompromised patients, while myelitis and large-vessel granulomatous arteritis are mainly seen with immunocompetent patients. Small-vessel encephalitis is the most common complication of VZV, but typically takes weeks to months from infection onset to present as chronic progressive encephalitis. It tends to develop without an antecedent rash, which is in stark contrast to large-vessel encephalitis. The finding of either VZV antibody or viral DNA in cerebrospinal fluid is strong evidence for the diagnosis of small-vessel VZV encephalitis [12]. Small-vessel encephalitis is life-threatening, and death from this disease is common. On the other hand, large-vessel encephalitis is typically characterized by having an acute focal deficit, such as a stroke. Both small and large vessel encephalitis occur infrequently. The type of encephalitis was not identified or documented in our patient. As mentioned above, Gilden et al. propose that VZV encephalitis may be a vasculopathy, but the manner of VZV resulting in development of encephalitis is not understood [12,13]. Overall, treatment for the complication of VZV encephalitis calls for an extended course of acyclovir [12,13]. 

The recommended treatment for VZV encephalitis is acyclovir. Acyclovir inhibits the viral DNA polymerase enzyme, incorporating into viral DNA and resulting in chain termination [14]. DNA of the host cell is minimally affected, making acyclovir highly selective for inhibiting the replication of herpesvirus DNA [14]. Gilden et al. recommend treatment for small-vessel encephalitis with acyclovir 15 to 30 mg a day for 10 days or longer if the patient is severely immunocompromised [12]. The recommended treatment by Gilden et al. for large-vessel encephalitis is intravenous acyclovir 10 to 15 mg per kg of body weight, three times a day for seven to 10 days, with the addition of a short course of corticosteroids to reduce inflammation [12]. Another treatment regimen in the literature is a 14-day course of intravenous acyclovir, potentially with a course of oral corticosteroids for seven days [13]. 

Giehl et al. recommend using acyclovir to treat patients with concurrent HSV and VZV reactivation at the dosage and course needed to manage VZV infections [6]. This treatment regimen appeared effective in the study and expedited the resolution of concurrent HSV and VZV skin lesions [6]. Our patient was started on intravenous acyclovir at renal-adjusted dosing of five mg/kg every 24 hours for a seven-day course, which was later extended to 10 days to adequately treat the encephalitis. He was also given topical mupirocin ointment to apply to areas with superficial crusting. Overall, treatment with acyclovir is most efficacious when initiated early in the presentation to reduce new lesion formation and acute pain [12,15]. 

## Conclusions

Simultaneous HSV reactivation and recurrent VZV infection are rare. Various disease states or trauma to the neurologic system may be the underlying trigger for the concurrent reactivation. A cutaneous eruption with suspicious lesions should prompt clinical and laboratory evaluation for numerous pathogens, in this case, multiple herpesviruses. This approach will allow for expedited diagnosis and management of both HSV and VZV and may prevent the evolution of more severe complications of infection.
